# P175: A new training and assessment support of knowledge in hand hygiene: CD - ROM type quiz prepared by the Department of Health Bizerte

**DOI:** 10.1186/2047-2994-2-S1-P175

**Published:** 2013-06-20

**Authors:** H Rhida, H Souilah, A Gzara, H Kammoun, I Dhaouadi

**Affiliations:** 1 Regional Directorate of Health Bizerte, Bizerte, Tunisia; 2 Kassab Orthopedic Institute, La Manouba, Tunisia; 3 Regional Directoion of Health, Tunis, Tunisia

## Introduction

The year 2010 has been very prolific in Tunisia with regard to educational materials and training on hand hygiene: brochures, pamphlets, fact sheets, slide shows, quizzes, ... All these documents were developed by teams of volunteers and designed inspired by the WHO documentation issued in 2009 (adoption after adaptation). They were gathered in a toolbox hand hygiene. The Department of Hygiene Bizerte participated in enriching the content of this toolkit for the development of a CD-ROM training and knowledge assessment of hand hygiene.

## Objectives

The development of this new medium should meet the need of healthcare workers and hygienists in training materials to'' hand hygiene adapted to new technologies and also to standardize and unify the concepts related to hand hygiene and harmonize methods and preservation techniques of hand hygiene and evaluation methods of knowledge on the subject.

## Methods

The development of this medium has used a multidisciplinary working group of volunteers. Validation of documents has been assigned to resource persons and experts in health care safety.

## Results

The CD-ROM has been designed for two types of use: learning and evaluation. It includes 5 sections: - Section I:'''' Test-Quiz with 50 questions, - Section II: Quiz'' -'' Training with 50 question and answer pairs, - Section III:'' To know'' consisting of a slideshow that can be used as a medium of animation training group (5 parts), - Section IV:'' hands'' other views, slideshow featuring illustrations from drawings by hand painting, - Section V: Hand'' across cultures'' section with proverbs and quotes in different languages on the utility of hand.

## Conclusion

Obviously, this is a first version that can’t claim to be complete and final, which will be followed by successive versions certainly enriched and improved by taking into account the reactions and feedback from users.

## Disclosure of interest

None declared

